# Sub-Inhibitory Concentrations of Chlorhexidine Induce Resistance to Chlorhexidine and Decrease Antibiotic Susceptibility in *Neisseria gonorrhoeae*

**DOI:** 10.3389/fmicb.2021.776909

**Published:** 2021-11-25

**Authors:** Jolein G. E. Laumen, Christophe Van Dijck, Sheeba S. Manoharan-Basil, Saïd Abdellati, Irith De Baetselier, Vicky Cuylaerts, Tessa De Block, Dorien Van den Bossche, Basil B. Xavier, Surbhi Malhotra-Kumar, Chris Kenyon

**Affiliations:** ^1^STI Unit, Department of Clinical Sciences, Institute of Tropical Medicine, Antwerp, Belgium; ^2^Laboratory of Medical Microbiology, Vaccine and Infectious Disease Institute, University of Antwerp, Antwerp, Belgium; ^3^Clinical Reference Laboratory, Department of Clinical Sciences, Institute of Tropical Medicine, Antwerp, Belgium; ^4^Department of Medicine, University of Cape Town, Cape Town, South Africa

**Keywords:** *Neisseria gonnorhoeae*, antimicrobial resistance, mouthwash, Listerine^®^, chlorhexidine, cross-resistance

## Abstract

**Objectives:** Chlorhexidine digluconate (chlorhexidine) and Listerine^®^ mouthwashes are being promoted as alternative treatment options to prevent the emergence of antimicrobial resistance in *Neisseria gonorrhoeae*. We performed *in vitro* challenge experiments to assess induction and evolution of resistance to these two mouthwashes and potential cross-resistance to other antimicrobials.

**Methods:** A customized morbidostat was used to subject *N. gonorrhoeae* reference strain WHO-F to dynamically sustained Listerine^®^ or chlorhexidine pressure for 18 days and 40 days, respectively. Cultures were sampled twice a week and minimal inhibitory concentrations (MICs) of Listerine^®^, chlorhexidine, ceftriaxone, ciprofloxacin, cefixime and azithromycin were determined using the agar dilution method. Isolates with an increased MIC for Listerine^®^ or chlorhexidine were subjected to whole genome sequencing to track the evolution of resistance.

**Results:** We were unable to increase MICs for Listerine^®^. Three out of five cultures developed a 10-fold increase in chlorhexidine MIC within 40 days compared to baseline (from 2 to 20 mg/L). Increases in chlorhexidine MIC were positively associated with increases in the MICs of azithromycin and ciprofloxacin. Low-to-higher-level chlorhexidine resistance (2–20 mg/L) was associated with mutations in NorM. Higher-level resistance (20 mg/L) was temporally associated with mutations upstream of the MtrCDE efflux pump repressor (*mtrR*) and the *mlaA* gene, part of the maintenance of lipid asymmetry (Mla) system.

**Conclusion:** Exposure to sub-lethal chlorhexidine concentrations may not only enhance resistance to chlorhexidine itself but also cross-resistance to other antibiotics in *N. gonorrhoeae*. This raises concern regarding the widespread use of chlorhexidine as an oral antiseptic, for example in the field of dentistry.

## Introduction

The ongoing emergence of antimicrobial resistance (AMR) in *Neisseria gonorrhoeae* has motivated research into antibiotic-sparing treatment options to treat this pathogen (Chow et al., 2021; Van Dijck et al., 2021b). These have included the use of antiseptic mouthwashes to prevent and treat oropharyngeal infection with *N. gonorrhoeae* (Chow et al., 2017, 2020, 2021; Van Dijck et al., 2021a,b). The oropharynx is thought to play an important role in both the transmission of *N. gonorrhoeae* and the acquisition of AMR (Lewis, 2015). This is related to a number of factors, including poor antimicrobial penetration and horizontal gene transfer of AMR from commensal *Neisseria* at this site (Lewis, 2015; Fiore et al., 2020).

Two mouthwashes have been evaluated in clinical and *in vitro* studies thus far – Listerine^®^ and chlorhexidine digluconate. Listerine Cool Mint^®^ (henceforth termed Listerine^®^) is a bactericidal mouthwash containing three essential oils [eucalyptol (0.092%); menthol (0.042%) and thymol (0.064%)], methyl salicylate (0.060%) and ethanol (21.6%) (Supplementary Material 1). It exerts its bactericidal effect via a number of pathways, including disruption of the cytoplasmic membranes (Faleiro, 2011; Nazzaro et al., 2013). *In vitro*, Listerine^®^ was effective against *N. gonorrhoeae* and in a clinical trial, a single Listerine^®^ mouthwash and gargle reduced pharyngeal culture positivity by 84% (Chow et al., 2017). Results from subsequent studies suggest that this effect is transient. Two large randomized controlled trials have found that the regular use of Listerine^®^ in men having sex with men does not reduce the incidence of *N. gonorrhoeae* or other STIs (Chow et al., 2021; Van Dijck et al., 2021b). Resistance to essential oils has been reported in several bacteria but never in *N. gonorrhoeae*. In *Staphylococcus aureus*, resistance to *Melaleuca alternifolia* (tea tree oil) has been detected (Nelson, 2000). After exposure to sub-inhibitory concentrations of the essential oils *Leptospermum scoparium* (manuka), *Origanum majorana* (marjoram) and *Origanum vulgare* (oregano), *S. aureus* acquired resistance to a wide range of antibiotics including ampicillin, erythromycin, neomycin and sulfamethoxazole (Turchi et al., 2019). In *Salmonella* s*enftenberg*, exposure to the basil oil component, linalool, resulted in adaptation to the basil oil mixture, as well as cross resistance to the antibiotics trimethoprim, sulfamethoxazole, chloramphenicol and tetracycline - increasing their MICs by 2- to 32- fold (Kalily et al., 2017). At least one group of authors have claimed that Listerine does not induce resistance to essential oils but provided little experimental evidence to back this claim up (Hughes and Dean, 2016).

Chlorhexidine digluconate (henceforth termed chlorhexidine) is widely regarded as the gold standard oral antiseptic (Balagopal and Arjunkumar, 2013). Its antibacterial mechanism of action is based on damaging the bacterial cytoplasmic membrane and subsequent leakage of cytoplasmic components (Cieplik et al., 2019). *In vitro* studies have established that *N. gonorrhoeae* is highly susceptible to killing by chlorhexidine (Rabe and Hillier, 2000; Victoria et al., 2018; Van Dijck et al., 2020). However, a recent clinical trial was ended early since twice daily gargling with chlorhexidine for six days failed to eradicate *N. gonorrhoeae* in the oropharynx (Van Dijck et al., 2021a).

To the best of our knowledge, decreased susceptibility to chlorhexidine has never been reported in *N. gonorrhoeae* before. It has, however, been reported in several other bacteria including *Enterobacter* spp., *Pseudomonas* spp., *Proteus* spp., *Providencia* spp., *Enterococcus* spp., and an array of oral bacterial species (Emilson and Fornell, 1976; Wade and Addy, 1989; Kampf, 2016; Kitagawa et al., 2016; Wang et al., 2017). One of the mechanisms responsible for chlorhexidine resistance is alteration of bacterial cell membranes (Kampf, 2018; Cieplik et al., 2019).

For example, in *Pseudomonas aeruginosa* and *P. stutzeri*, alterations in the outer membrane and lipopolysaccharide profiles can act as a barrier to prevent chlorhexidine from entering the cell (Tattawasart et al., 2000; Guérin-Méchin et al., 2004). Upregulation of efflux pumps has also been found to confer resistance to chlorhexidine. For example, upregulation of the resistance-nodulation-division (RND) efflux protein, AdeABC, resulted in reduced chlorhexidine susceptibility in *Acinetobacter baumannii* and *Escherichia coli* (Hassan et al., 2013; Kampf, 2018; Cieplik et al., 2019). Upregulation of RND pumps results in resistance to a number of clinically important bacteria-antimicrobial combinations (Maris, 1991; Knapp et al., 2013; Fernández-Cuenca et al., 2015). In *Klebsiella pneumoniae*, for example, exposure to chlorhexidine led to resistance to the last-resort antibiotic, colistin, mediated by the major facilitator superfamily (MFS) efflux pump gene (Wand et al., 2017).

The use of antiseptic mouthwashes remains widespread (Walker et al., 2016). A representative sample of residents in the Grampian region of Scotland, for example, found that 62% reported using mouthwash, and 25.1% did so daily (Macfarlane et al., 2010). These considerations provided the motivation for the current study where we assessed if resistance to chlorhexidine and Listerine^®^ could be induced in *N. gonorrhoeae*. We also evaluated if these antiseptics could induce cross-resistance to important antibiotics. Currently, *N. gonorrhoeae* infection is treated with ceftriaxone mono therapy, or dual therapy with ceftriaxone and azithromycin (Cyr et al., 2020; Unemo et al., 2020). An important alternative potential treatment option, when susceptibility has been confirmed, is ciprofloxacin (Klausner et al., 2021).

## Materials and Methods

### Experimental Procedures

We exposed *N. gonorrhoeae* reference strain WHO-F to increasing concentrations of Listerine Cool Mint^®^ (Johnson & Johnson, New Brunswick, NH, United States) and Corsodyl containing chlorhexidine (Supplementary Material 1) using a continuous-culture device known as a morbidostat (Toprak et al., 2013; Verhoeven et al., 2019). According to the EUCAST (v11.0) breakpoints for antimicrobial susceptibility, WHO-F is susceptible to azithromycin, cefixime, ceftriaxone and ciprofloxacin with minimal inhibitory concentrations (MICs) of 0.125, <0.016, <0.002, and 0.004 mg/L, respectively^1^ (Unemo et al., 2016).

The morbidostat was built following the detailed instructions by Toprak et al., modified for *N. gonorrhoeae* according to Toprak et al. (2013), Verhoeven et al. (2019). The experiment was carried out exactly as previously reported in the context of a *N*. *gonorrhoeae* morbidostat experiment selecting for azithromycin resistance in our laboratory (Laumen et al., 2021). In brief, *N. gonorrhoeae* cultures were put under sustained Listerine^®^ (*n* = 6) and chlorhexidine pressure (*n* = 5), whereas two negative control cultures where exclusively exposed to gonococcal (GC) broth supplemented with 1% IsoVitaleX (BD BBLTM) and vancomycin, colistin, nystatin and trimethoprim selective supplement (VCNT), hereafter called GC medium. GC broth contained per liter, 15 g of proteose peptone 3 (Carl Roth, Karlsruhe), 1 g of soluble starch, 4 g of K_2_HPO_4_, 1 g of KH_2_PO_4_ and 5 g of NaCl supplemented with 1% IsoVitaleX (BBL). The initial concentrations used in these experiments were based on early *in vitro* experiments whereby the growth of *N. gonorrhoeae* was investigated after 60 min of exposure to ten-fold dilutions of the mouthwashes. The highest mouthwash concentration that resulted in a lawn of *N. gonorrhoeae* growth after inoculation, comparable to the control plate where no mouthwash was added, was used as a starting dilution of the mouthwash.

The initial chlorhexidine concentration used was 0.2 mg/L and was increased twice weekly until a concentration of 80 mg/L was attained in the morbidostat reservoir. The Listerine^®^ concentration was increased in steps from a 100-fold to two-fold dilution. The antiseptics were diluted in GC medium.

### Sampling and Agar Dilution

Twice a week, culture suspensions were inoculated on blood agar plates and incubated for 24 h at 36.5°C and 5-7% CO_2_. Cultures were checked for purity and transferred in 1 mL of skim milk supplemented with 20% glycerol and stored at –80°C. Once the morbidostat experiment was terminated, stored samples were cultured, a single colony was taken and used for MIC determination and whole genome sequencing.

Minimal inhibitory concentrations (MICs) were determined using agar dilution for Listerine^®^, chlorhexidine, azithromycin, cefixime, ceftriaxone and ciprofloxacin according to the guidelines of CLSI (Wayne, 2020). Isolates with an increase in MIC for Listerine^®^ or chlorhexidine were subjected to WGS, as were an isolate from the previous, first and last sampling occasion of the same morbidostat culture.

Spearman’s correlation was used to determine the correlation between the MIC of each mouthwash and the MIC of azithromycin, cefixime, ceftriaxone and ciprofloxacin. Only the first sampling timepoint and each timepoint the MICs of the mouthwashes increased were included for this analysis.

### Whole Genome Sequencing and Analysis

Genomic DNA was isolated using MasterPure complete DNA and RNA purification kit (Epicenter, United States) according to the manufacturer’s instructions. The DNA concentration was assessed using the Qubit ds DNA HS Assay Kit in a Qubit Fluorometer 3.0 (Thermo Fisher Scientific, United States). Whole genome sequencing of clones was performed via 2 × 250 bp paired end sequencing (Nextera XT sample) preparation kit and Miseq, Illumina Inc United States.

Genetic changes facilitated by the mouthwashes were explored by comparing whole genome sequences against the reference genome.

Reads were mapped against the reference WHO-F (NZ_LT591897) and variants were extracted using the CLC Genomics Workbench v20 (CLC Bio, Cambridge, MA, United States). Variants were manually examined to establish true variants, which were consequently confirmed by *de novo* assemblies. In short, Shovill (v1.0.4) (Seemann, 2019) was used for assembly which uses SPAdes (v3.14.0) (Prjibelski et al., 2020) with the following parameters: –trim–depth 150–opts “–cov-cutoff auto –careful.” The quality of the contigs were verified with Quast (v5.0.2) (Gurevich et al., 2013) followed by annotation using Prokka (v1.14.6) (Seemann, 2014). All the sequences generated in this study were submitted to Bioproject PRJNA756860.

## Results

### Phenotypic Susceptibility Assessment of *Neisseria gonorrhoeae* Isolates Following Listerine^®^ Exposure

All six cultures exposed to Listerine^®^ lost viability within 18 days after the start of the experiment. This occurred right after the Listerine^®^ concentration in the reservoir was increased from a 5-fold to a 2-fold dilution. The Listerine^®^ MICs did not increase during the course of the experiment (Supplementary Material 2).

### Phenotypic Susceptibility Assessment of *Neisseria gonorrhoeae* Isolates Following Chlorhexidine Exposure

**FIGURE 1:**
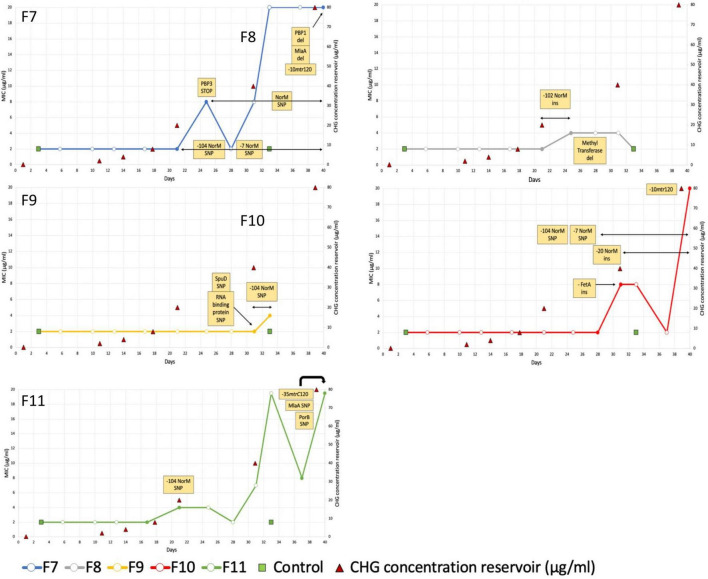
Minimal Inhibitory Concentration of 5 *Neisseria gonorrhoeae* WHO-F cultures under chlorhexidine selection pressure over time. Filled circles depict the timepoints that were sequenced. Variants observed in WGS data are indicated in the yellow boxes. Based on these data, the duration of their detection is depicted via black arrows.

For the five cultures under chlorhexidine selection pressure, MICs of three cultures increased 10-fold (From 2 mg/L at baseline to 20 mg/L after 40 days of exposure) (Figure 1: F7, F10 and F11). For two other cultures, F8 and F9, exposure to GC medium with a chlorhexidine concentration of 20 mg/L added from day 21 revealed initial decreased growth (data not shown) and subsequent no growth after 37 days. The MICs of these cultures increased 4-fold from 2 to 8 mg/L (Figure 1: F8 and F9). The MICs of the two negative control cultures remained 2 mg/L.

The MICs of azithromycin, cefixime, ceftriaxone and ciprofloxacin increased from 0.125, 0.002, <0.001 and 0.004 mg/L to 0.5, 0.004, 0.002 and 0.06 mg/L, respectively (Supplementary Material 2). The MICs of azithromycin and ciprofloxacin correlated positively with the chlorhexidine MIC: *r*_s_ = 0.743 (*p* = 0.003); *r*_s_ = 0.620 *(p* = 0.018), respectively.

### Whole-Genome Sequencing Analysis of Chlorhexidine-Adapted Strains

**FIGURE 2:**
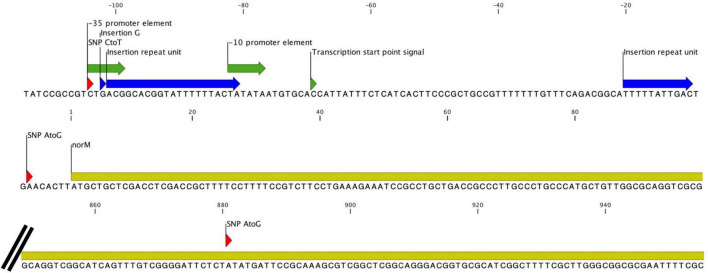
All variants observed in the *norM* gene and its promoter. Insertions are indicated in blue while SNPs are indicated in red. The coding region of the gene is colored yellow, and the promoter elements are green.

All five cultures developed variants in the gene encoding the multidrug efflux MATE transporter NorM (Figure 1 and Table 1). In total, six different variants were observed related to the *norM* gene: a single nucleotide polymorphism (SNP), a one base pair (bp) insertion and a 22-bp repeat unit within the putative –35 promoter element IV, a SNP and a 11-bp repeat unit at the putative ribosome binding site (RBS) and a SNP in the coding region (Figures 1, 2 and Table 1). In all except one culture (F8), a SNP in the putative –35 promoter element was observed, and in most cases this SNP arose at the timepoint before the MIC started to increase and remained present when a MIC of 20 mg/L was reached (Figure 1; F7, F10 and F11). In two of these cultures, F7 and F10, an additional SNP at the RBS was observed at the same timepoints. Once the MIC increased to 8 and 20 mg/L, either the repeat insertion at the RBS or the SNP in the NorM encoding region occurred.

**TABLE 1:** Variants occurring in at least 2 cultures of *N.gonorrhoeae* strain WHO-F exposed to chlorhexidine in the morbidostat set-up.

	Locus tag	Variants in WHO-F cultures by vial number				
Gene	WHO-F NZ_LT591897	F7	F8	F9	F10	F11
5′ UTR *mtrR*	C7S05_RS08275	G131A*(Ohneck et al., 2011)			G131A*	delA57*(Hagman and Shafer, 1995)
(5′ UTR) *norM*	C7S05_RS02320	C104T*(Rouquette-Loughlin et al., 2003), Tyr294Cys, A7G*(Rouquette-Loughlin et al., 2003)	Ins(GACGGCAC GGTATTTTTTA aCTA)102*	C104T*	C104T*, A7G*, insTTTTTATTG AC20*	C104T*
*mlaA*	C7S05_RS13185	Asn122del				Pro144Ser

*Ins: insertion del: deletion UTR: Untranslated Region.*

* Indicates the number of base pairs upstream of the translated region. Variants located within the UTR are described as nucleotide changes, variants in the translated region are described as amino acid changes. The numbers in superscript are references to previous studies that have found these same mutations in N. gonorrhoeae.

All isolates with a MIC of 20 mg/L (10-fold increase compared to baseline), carried variants in the promoter of the MtrCDE multidrug efflux pump (Figure 1 and Table 1). In cultures F7 and F10, a C-to-T transition mutation 120 bp upstream of the *mtrC* start codon (mtr120) was observed, resulting in a consensus –10 element generating a novel promoter for MtrCDE transcription (Ohneck et al., 2011). Culture F11 acquired a single base pair deletion within the 13-bp inverted repeat sequence in the promoter of the MtrCDE repressor MtrR (Hagman and Shafer, 1995).

In addition, variants in the *mlaA* gene, part of the maintenance of lipid asymmetry (Mla) system were found in two of the three isolates with a MIC of 20 mg/L (Figure 1 and Table 1). In culture F7, asparagine at position 122 was deleted whilst in culture F11 a transition of proline to serine was detected at position 144.

A comprehensive list of all variants detected is provided in Supplementary Material 3. Variants found in only one culture included two penicillin binding proteins (PBP1a and PBP3, culture F7), an outer membrane porin (PorB, culture F11) and protein (FetA, culture F10) and an ABC transporter substrate-binding protein (SpuD, culture F9).

## Discussion

We report the first experimental evidence that sustained chlorhexidine exposure can result in reduced susceptibility to chlorhexidine and other antimicrobials in *N. gonorrhoeae.* In our experiments, the first step in this pathway involved mutations in the gene encoding NorM.

The NorM protein is a Na^+^-drug antiporter and member of the multidrug and toxic compound extrusion (MATE) family (Kuroda and Tsuchiya, 2009). Two point mutations upstream of the *norM* gene have previously been reported to result in increased expression of *norM* and hence decreased gonococcal susceptibility to several cationic compounds and ciprofloxacin (Rouquette-Loughlin et al., 2003; Sánchez-Busó et al., 2021). These two variants were also observed in this current study: a C-to-T mutation in the putative –35 promoter element and an A-to-G mutation 7 bp upstream of the ATG codon resulting in an alteration of the putative RBS. In *N. gonorrhoeae* strain FA19, these mutations increased the MIC for ciprofloxacin 2 to 4-fold (Rouquette-Loughlin et al., 2003; Golparian et al., 2014). In our experiments, two isolates acquired both mutations in the absence of any other variants. The MIC for ciprofloxacin increased 2-fold in one of these isolates but did not increase in the other isolate. In both cases, the MICs for chlorhexidine remained similar to baseline. Besides other variants (insertion and repeat units) within the putative –35 promoter element and the RBS, one SNP was found in the coding region of *norM*. The tyrosine to cysteine substitution at position 294 is located at the cation-binding site.

Previous studies have found that sodium ion binding at the cation-binding site causes a conformational rearrangement which in turn leads to the disruption of the protein-drug interactions and triggers the extrusion of the bound drug into the periplasmic space (Lu et al., 2013; Leung et al., 2014). Residue 294 plays an important role in the stabilization of the sodium ion at the binding site, which is crucial for the subsequent drug extrusion stage (Supplementary Material 4) (Leung et al., 2014). Further experimental work will be required to assess the effect of this mutation.

Mutations in the mtrCDE efflux pump were also prominent. In all three cultures that showed a 10-fold increase in chlorhexidine MIC, a variant in the region between the mtrCDE efflux pump and its repressor MtrR was observed. In cultures F7 and F10 (Figure 1 and Supplementary Table 1) this was the well-known SNP 120bp upstream of the *mtrC* start codon, resulting in a consensus –10 element generating a novel promoter for *mtrCDE* transcription (Warner et al., 2008; Ohneck et al., 2011). In culture F11, a single-base pair deletion in the inverted repeat sequence positioned within the *mtrR* promoter was found (Hagman and Shafer, 1995). This variant represses transcription of the repressor MtrR and hence enhances expression of *mtrC*. Since mutations in the MtrCDE efflux pump are well known determinants of resistance to tetracyclines, macrolides and cephalosporins, it is plausible that these mutations are responsible for the observed cross-resistance to these antimicrobial classes in our experiment (Unemo and Shafer, 2014).

Isolates with reduced susceptibility to chlorhexidine possessed mutations in *mlaA.* Similarly, mutations in the *mlaA* gene were previously identified in *N. gonorrhoeae* clones resistant to the quaternary ammonium surfactant dodecyl-prydinium bormide (C_12_PB) (Calado et al., 2021). The maintenance of lipid asymmetry system (Mla) is responsible for retrograde transport of phospholipids, which is crucial for maintaining and repairing the outer membrane of Gram-negative bacteria, including *N. gonorrhoeae* (Baarda et al., 2019). The asymmetry of this membrane provides a more effective barrier to toxic lipophilic, hydrophilic and amphipathic molecules than a phospholipid bilayer would be (Baarda et al., 2019). By maintaining the lipid asymmetry of outer membrane, MlaA, may thus play a role in defense against compounds such as chlorhexidine that work by disturbing membrane function. Deletion of the MlaA protein has been shown to disrupt membrane asymmetry and increase susceptibility to antibiotics that target the outer-membrane/cell wall in *N. gonorrhoeae* (Baarda et al., 2019).

MlaA may also affect chlorhexidine susceptibility via its role in membrane vesicle genesis. Experiments in *Porphyromonas gingivalis* have found that membrane vesicles could bind to chlorhexidine and thereby protect the bacteria (Grenier et al., 1995). In *N. gonorrhoeae*, *Haemophilus influenzae* and *Vibrio cholerae*, deletion of *mlaA* has been shown to increase the production of membrane vesicles *in vitro* (Roier et al., 2016; Baarda et al., 2019). A number of factors such as the availability of iron, presence of human defensins and anatomical site of infection in murines have been shown to influence the expression of *mlaA* (Baarda et al., 2019). The complexity of this system in addition to the recent emphasis on the role of outer membrane vesicles in antimicrobial resistance in *N. gonorrhoeae* suggests that further *in vivo* work will be required to establish the phenotypic effects of the mutations we found in *mlaA* (Supplementary Material 5) (Unitt et al., 2021).

Previous studies reported similar chlorhexidine MICs in *N. gonorrhoeae* as in the current study (range 2–8mg/L) (Waitkins and Geary, 1975; Rabe and Hillier, 2000; Victoria et al., 2018). Here, we were able to effect a 10-fold increase in chlorhexidine MIC and concomitant minor increases in azithromycin, ciprofloxacin and cefixime MICs. One of the major limitations of this study is that we have not experimentally confirmed that specific genetic variants result in a particular resistant phenotype. Furthermore, there are important differences in the exposure of *N. gonorrhoeae* to antiseptic agents between our *in vitro* model and what might transpire *in vivo*. The 10-fold increase in chlorhexidine MIC required a long period of exposure (33–40 days) and considerably lower concentrations of chlorhexidine (highest concentration 80 mg/L), than those used in clinical practice (typically 2000 mg/L). This important limitation means that our results are best conceptualized as a worst-case scenario. We cannot conclude from our results that even extensive clinical use of chlorhexidine would have an influence on antimicrobial susceptibility. On the other hand, *in vivo*, chlorhexidine concentrations and substantivity in the oral cavity can be affected by food, drinks, pH, saliva and serum (Waitkins and Geary, 1975; Rabe and Hillier, 2000; Tomás et al., 2010; Abouassi et al., 2014; Cieplik et al., 2019). Furthermore, chlorhexidine is unlikely to penetrate and kill *N. gonorrhoeae* localized within pharyngeal epithelial cells intracellularly or in crypts (Veien et al., 1976; Lewis, 2015; Chow et al., 2020). Its penetration into biofilms is also likely incomplete (Cieplik et al., 2019). These factors might create concentration gradients of chlorhexidine that could in turn select for resistance. Chlorhexidine may also select for resistance in commensal *Neisseria* species. This resistance could then be passed on to *N. gonorrhoeae* via transformation (Spratt et al., 1989; Ameyama et al., 2002). Anticipating worst-case scenarios is vital for species such as *N. gonorrhoeae* which are at risk of becoming untreatable with antibiotics (Unemo and Shafer, 2014).

Increasing doses of Listerine^®^ did not result in resistance to Listerine^®^ or cross-resistance to antibiotics. Our results suggest that Listerine^®^ may be a better option in this regard than chlorhexidine. One possible explanation for this difference between Listerine^®^ and chlorhexidine is the number of active ingredients – five in Listerine^®^ and one in chlorhexidine (Balagopal and Arjunkumar, 2013; Van Dijck et al., 2021b). Several targets need to be modified to hinder the effect of essential oils, which could reduce the potential of resistance induction (Yap et al., 2014). Overall, there is limited evidence from studies suggesting the spontaneous occurrence of essential oil resistance, and resistance to the combination of essential oils used in Listerine^®^ has never been reported (Yap et al., 2014). Gonococci remained highly susceptible even after prolonged exposure to sub-inhibitory concentrations, while *in vivo* an even two times higher concentration of Listerine is used than the final bactericidal dilution added in this experiment. A limitation of this study was the use of VCNT, which was added to the medium to prevent contamination (Verhoeven et al., 2019). It may be possible that Listerine^®^ increased the susceptibility of *N. gonorrhoeae* to VCNT.

If this occurred, it could have impeded the acquisition of mutants that conferred decreased susceptibility to Listerine^®^.

Although we cannot exclude this possibility, this study suggests that it is unlikely that resistance to Listerine will occur, making it an interesting antiseptic to use as a way to reduce selection pressure for gonococcal AMR (Chow et al., 2021; Van Dijck et al., 2021b). However, two large randomized controlled clinical trials recently showed that Listerine does not significantly reduce the incidence of oropharyngeal *N. gonorrhoeae* (Chow et al., 2021; Van Dijck et al., 2021b). On the other hand, we found that exposure to sub-lethal chlorhexidine concentrations may not only enhance resistance to chlorhexidine itself but also cross-resistance to other antibiotics. Intense exposure to chlorhexidine may result in resistance associated mutations in *N. gonorrhoeae* in three different efflux pumps: the MATE, RND and ABC transporters. We identified a number of mutations to assess in populations where the use of chlorhexidine is intense. In ventilated patients for example, chlorhexidine is used up to six times per day for the prevention of pneumonia (Hutchins et al., 2009). Mouthwashes containing chlorhexidine are used for over 40 years in the field of dentistry to treat gingivitis, periodontitis and tooth decay without being aware of the risk of bacterial resistance (Cieplik et al., 2019; Varoni et al., 2021). These results provide a cautionary note about the possible adverse effects of the excessive use of chlorhexidine. It may thus be prudent to restrict chlorhexidine mouthwash use to those indications with clear evidence of benefit.

## Data Availability Statement

The datasets presented in this study can be found in online repositories. The names of the repository/repositories and accession number(s) can be found in the article/Supplementary Material.

## Author Contributions

IDB, JGEL, and CK conceptualized the study. JGEL, CVD, SA, and BBX generated the laboratory results. JGEL, SSM-B, and TDB verified and analyzed the data. JGEL wrote the first draft of the manuscript. All authors reviewed and approved the final manuscript.

## Conflict of Interest

The authors declare that the research was conducted in the absence of any commercial or financial relationships that could be construed as a potential conflict of interest.

## Publisher’s Note

All claims expressed in this article are solely those of the authors and do not necessarily represent those of their affiliated organizations, or those of the publisher, the editors and the reviewers. Any product that may be evaluated in this article, or claim that may be made by its manufacturer, is not guaranteed or endorsed by the publisher.

## Footnotes

1
www.eucast.org
