# Supplementary material for: Sub-Inhibitory Concentrations of Chlorhexidine Induce Resistance to Chlorhexidine and Decrease Antibiotic Susceptibility in Neisseria gonorrhoeae
Source: Front Microbiol. 2021 Nov 25;12:776909. doi: 10.3389/fmicb.2021.776909 (PMC8660576; doi:10.3389/fmicb.2021.776909)
Supplement: Supplementary file 1 [file Table_1.DOCX]

**Supplementary table**

**The essential amino acids profile of CSM (% dry weight)**

|  | **Amino acids** | Asp | The | Val | Met | Ile | Leu | Phe | His | Arg | Lys |  |
| --- | --- | --- | --- | --- | --- | --- | --- | --- | --- | --- | --- | --- |
|  | **% dry weight** | 5.81 | 2.04 | 2.59 | 0.85 | 2.11 | 3.73 | 3.85 | 1.96 | 7.55 | 2.46 |  |
