# Supplementary material for: Sub-Inhibitory Concentrations of Chlorhexidine Induce Resistance to Chlorhexidine and Decrease Antibiotic Susceptibility in Neisseria gonorrhoeae
Source: Front Microbiol. 2021 Nov 25;12:776909. doi: 10.3389/fmicb.2021.776909 (PMC8660576; doi:10.3389/fmicb.2021.776909)
Supplement: Supplementary file 3 [file Table_3.DOCX]

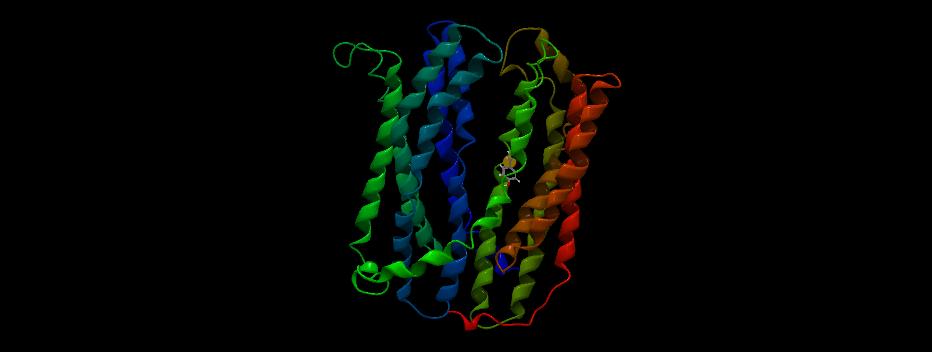


TM 8

A

B

C


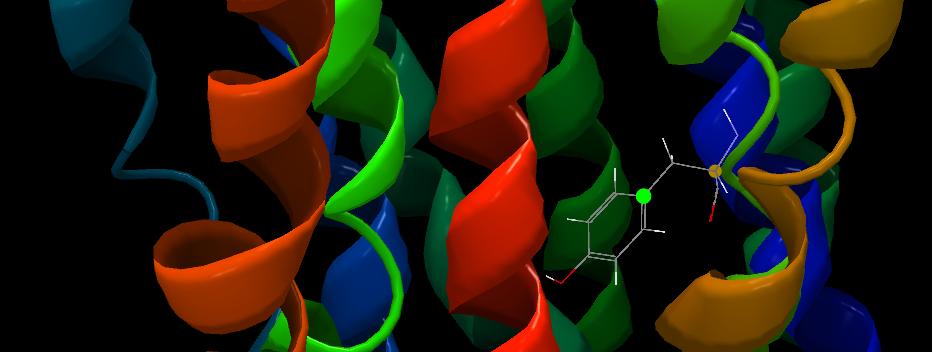


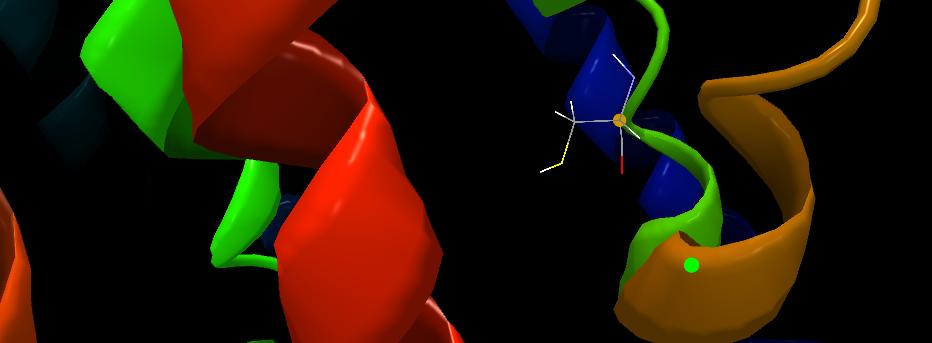
Supplement 4. (A) Structure of the NorM wild-type complex: V-shaped channel formed by transmembrane helices. The arrow indicates transmembrane (TM) 8, where position 294 is located. (B) NorM wild type with a tyrosine at position 294 located in transmembrane 8 helix. (C) NorM with a variant causing a tyrosine to cysteine change at position 294. The protein was modelled using The Phyre2 web portal for protein modelling, prediction and analysis.^1^ The graphics were produced with CLC Genomics Workbench version 20 (CLC Bio, Cambride, MA, USA).

1. Kelley LA, Mezulis S, Yates CM, Wass MN, Sternberg MJ. The Phyre2 web portal for protein modeling, prediction and analysis. *Nat Protoc* 2016; **10**: 845–58. Available at: http://dx.doi.org/10.1038/nprot.2015-053.
