# Supplementary material for: Sub-Inhibitory Concentrations of Chlorhexidine Induce Resistance to Chlorhexidine and Decrease Antibiotic Susceptibility in Neisseria gonorrhoeae
Source: Front Microbiol. 2021 Nov 25;12:776909. doi: 10.3389/fmicb.2021.776909 (PMC8660576; doi:10.3389/fmicb.2021.776909)
Supplement: Supplementary file 4 [file Table_4.DOCX]

B

D

A

C


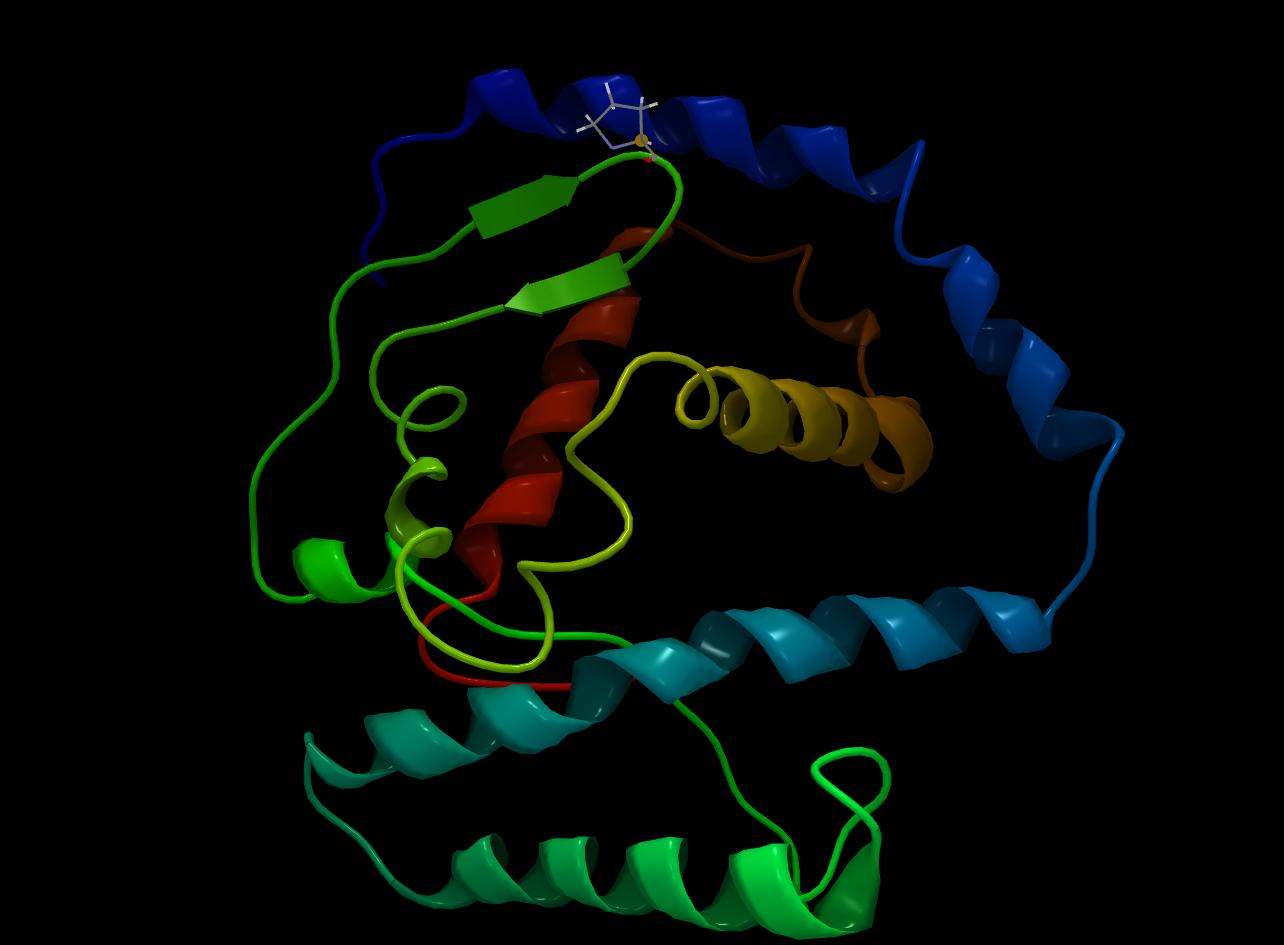

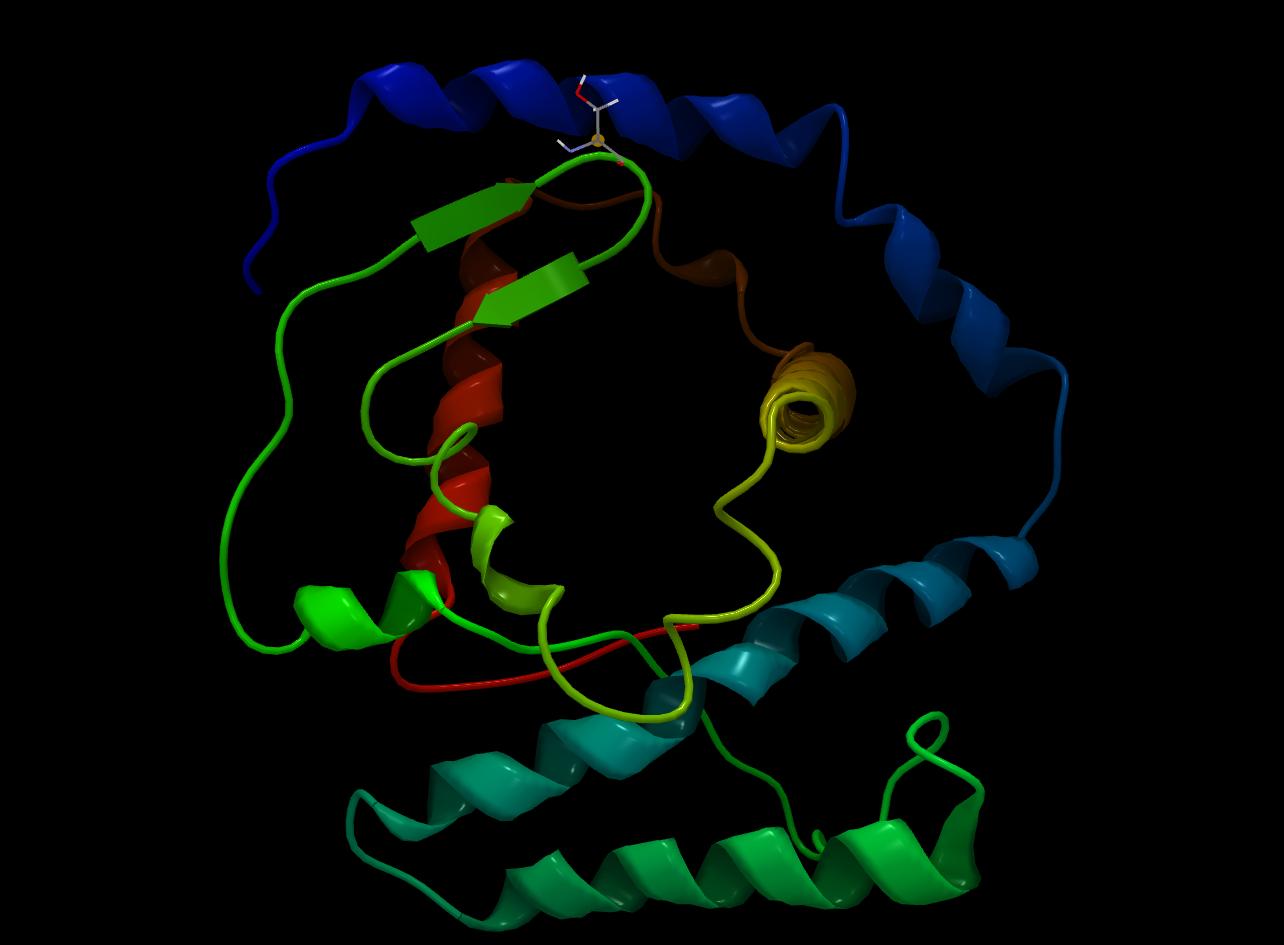


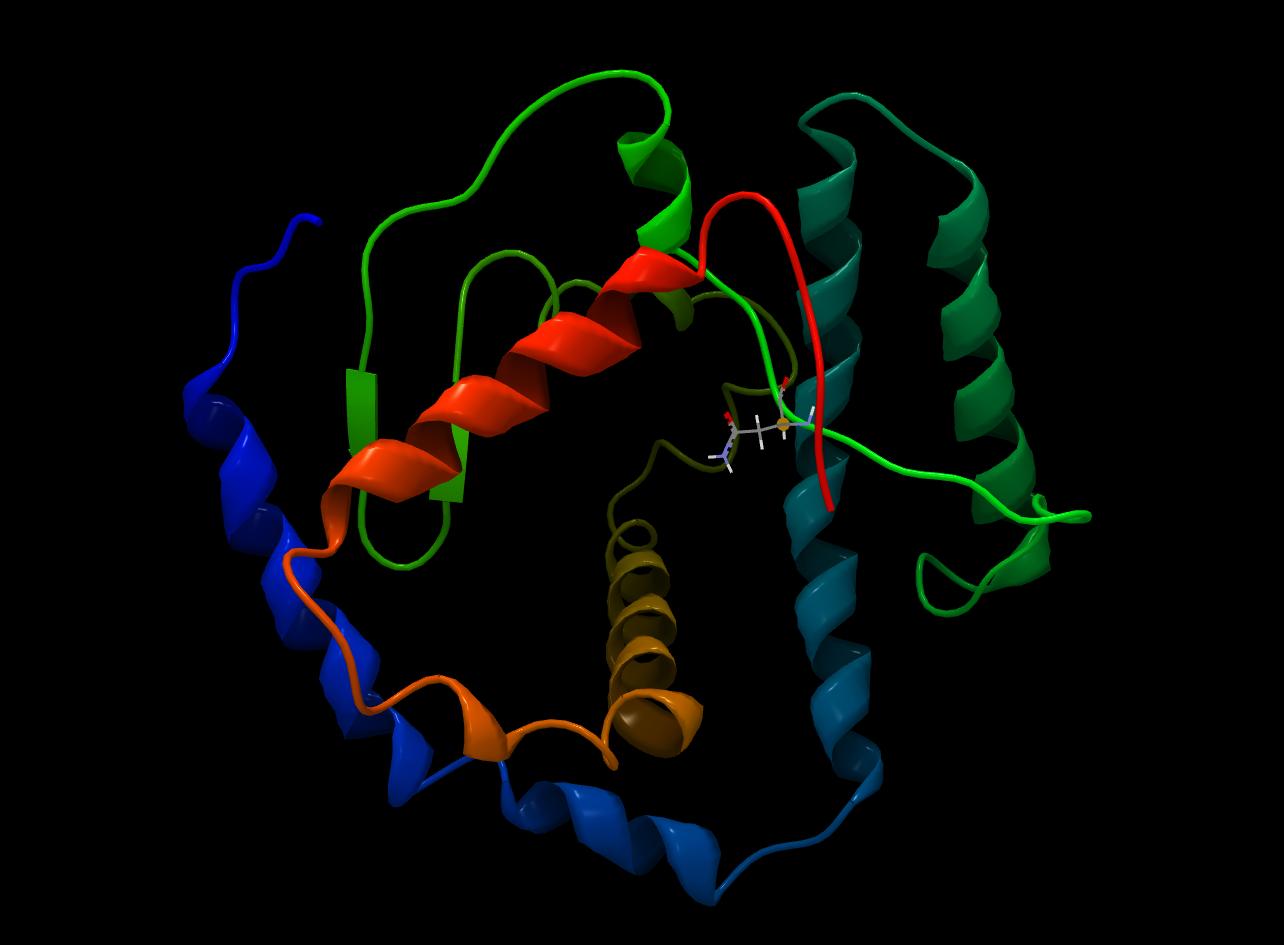

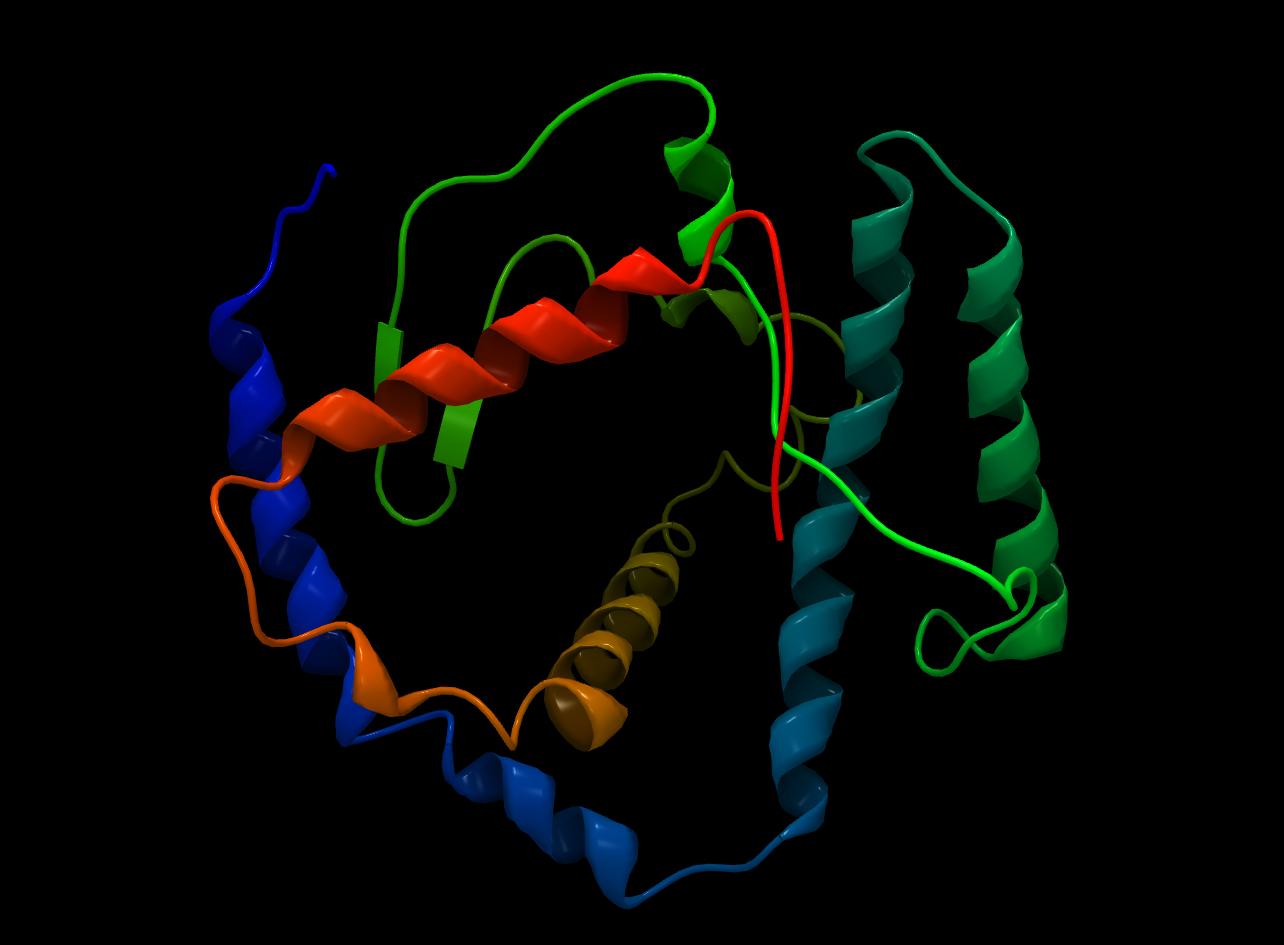


Supplement 5. (A) MlaA wild-type protein structure with a white circle indicating a proline at position 144, while (B) indicates a variant causing a proline to serine change at this position. (C) MlaA wild-type protein structure with a white circle indicating an asparagine at position 122. (D) MlaA protein structure with a deletion of the asparagine at position 122. The protein was modelled using The Phyre2 web portal for protein modeling, prediction and analysis.^1^ The graphics were produced with CLC Genomics Workbench version 20 (CLC Bio, Cambride, MA, USA).

1. Kelley LA, Mezulis S, Yates CM, Wass MN, Sternberg MJ. The Phyre2 web portal for protein modeling, prediction and analysis. *Nat Protoc* 2016; **10**: 845–58. Available at: http://dx.doi.org/10.1038/nprot.2015-053.
