# Supplementary material for: Sub-Inhibitory Concentrations of Chlorhexidine Induce Resistance to Chlorhexidine and Decrease Antibiotic Susceptibility in Neisseria gonorrhoeae
Source: Front Microbiol. 2021 Nov 25;12:776909. doi: 10.3389/fmicb.2021.776909 (PMC8660576; doi:10.3389/fmicb.2021.776909)
Supplement: Supplementary file 5 [file Table_5.DOCX]

Supplemental Table 2. Minimal inhibitory Concentrations to the mouthwashes and antibiotics, tested for all cultures at all timepoints.

| **Strain ID** | **Mouthwash experiment** | **Time after start (days)** | **Listerine MIC (µg/ml)** | **Chlorhexidine MIC (µg/ml)** | **Ceftriaxone MIC (µg/ml)** | **Ciprofloxacin MIC (µg/ml)** | **Cefixime MIC (µg/ml)** | **Azithromycin MIC (µg/ml)** |
| --- | --- | --- | --- | --- | --- | --- | --- | --- |
| F1 | Listerine | 3 | 25x diluted | 2 | < 0.001 | 0.004 | 0.002 | 0.125 |
| F1 | Listerine | 5,8 | 25x diluted | 2 | < 0.001 | 0.004 | 0.002 | 0.125 |
| F1 | Listerine | 10 | 25x diluted | 2 | < 0.001 | 0.004 | 0.002 | 0.125 |
| F1 | Listerine | 12,8 | 25x diluted | 2 | < 0.001 | 0.004 | 0.002 | 0.125 |
| F1 | Listerine | 16,8 | 25x diluted | 2 | < 0.001 | 0.004 | 0.004 | 0.125 |
| F2 | Listerine | 3 | 25x diluted | 2 | < 0.001 | 0.004 | 0.002 | 0.125 |
| F2 | Listerine | 5,8 | 25x diluted | 2 | < 0.001 | 0.004 | 0.004 | 0.125 |
| F2 | Listerine | 10 | 25x diluted | 2 | < 0.001 | 0.004 | 0.002 | 0.125 |
| F2 | Listerine | 12,8 | 25x diluted | 2 | < 0.001 | 0.004 | 0.002 | 0.125 |
| F2 | Listerine | 16,8 | 25x diluted | 2 | < 0.001 | 0.004 | 0.004 | 0.125 |
| F3 | Listerine | 3 | 25x diluted | 2 | < 0.001 | 0.004 | 0.002 | 0.125 |
| F3 | Listerine | 5,8 | 25x diluted | 2 | < 0.001 | 0.004 | 0.004 | 0.125 |
| F3 | Listerine | 10 | 25x diluted | 2 | < 0.001 | 0.004 | 0.004 | 0.125 |
| F3 | Listerine | 12,8 | 25x diluted | 2 | < 0.001 | 0.004 | 0.002 | 0.125 |
| F3 | Listerine | 16,8 | 25x diluted | 2 | < 0.001 | 0.004 | 0.002 | 0.125 |
| F4 | Listerine | 3 | 25x diluted | 2 | < 0.001 | 0.004 | 0.004 | 0.125 |
| F4 | Listerine | 5,8 | 25x diluted | 2 | < 0.001 | 0.004 | 0.004 | 0.125 |
| F4 | Listerine | 10 | 25x diluted | 2 | < 0.001 | 0.004 | 0.002 | 0.125 |
| F4 | Listerine | 12,8 | 25x diluted | 2 | < 0.001 | 0.004 | 0.002 | 0.125 |
| F4 | Listerine | 16,8 | 25x diluted | 2 | < 0.001 | 0.004 | 0.002 | 0.125 |
| F5 | Listerine | 3 | 25x diluted | 2 | < 0.001 | 0.004 | 0.002 | 0.125 |
| F5 | Listerine | 5,8 | 25x diluted | 2 | < 0.001 | 0.004 | 0.002 | 0.125 |
| F5 | Listerine | 10 | 25x diluted | 2 | < 0.001 | 0.004 | 0.002 | 0.125 |
| F5 | Listerine | 12,8 | 25x diluted | 2 | < 0.001 | 0.004 | 0.002 | 0.125 |
| F5 | Listerine | 21 | 25x diluted | 2 | < 0.001 | 0.004 | 0.004 | 0.25 |
| F6 | Listerine | 3 | 25x diluted | 2 | < 0.001 | 0.004 | 0.002 | 0.125 |
| F6 | Listerine | 5,8 | 25x diluted | 2 | < 0.001 | 0.004 | 0.002 | 0.125 |
| F6 | Listerine | 10 | 25x diluted | 2 | < 0.001 | 0.004 | 0.002 | 0.125 |
| F6 | Listerine | 12,8 | 25x diluted | 2 | < 0.001 | 0.004 | 0.004 | 0.125 |
| F6 | Listerine | 16,8 | 25x diluted | 2 | < 0.001 | 0.004 | 0.004 | 0.125 |
| F7* | Chlorhexidine | 3 | 25x diluted | 2 | < 0.001 | 0.004 | 0.002 | 0.125 |
| F7 | Chlorhexidine | 5,8 | 25x diluted | 2 | < 0.001 | 0.004 | 0.002 | 0.125 |
| F7 | Chlorhexidine | 10 | 25x diluted | 2 | < 0.001 | 0.004 | 0.002 | 0.125 |
| F7 | Chlorhexidine | 12,8 | 25x diluted | 2 | < 0.001 | 0.004 | 0.004 | 0.125 |
| F7 | Chlorhexidine | 16,8 | 25x diluted | 2 | < 0.001 | 0.004 | 0.002 | 0.25 |
| F7* | Chlorhexidine | 21 | 25x diluted | 2 | < 0.001 | 0.008 | 0.002 | 0.125 |
| F7* | Chlorhexidine | 24,8 | 25x diluted | 8 | < 0.001 | 0.004 | 0.002 | 0.125 |
| F7 | Chlorhexidine | 28 | 25x diluted | 2 | < 0.001 | 0.004 | 0.002 | 0.125 |
| F7 | Chlorhexidine | 31 | 25x diluted | 8 | < 0.001 | 0.004 | 0.002 | 0.125 |
| F7 | Chlorhexidine | 33 | 25x diluted | 20 | < 0.001 | 0.004 | 0.002 | 0.5 |
| F7 | Chlorhexidine | 37 | 25x diluted | 20 | < 0.001 | 0.004 | 0.004 | 0.5 |
| F7* | Chlorhexidine | 40 | 25x diluted | 20 | < 0.001 | 0.004 | 0.004 | 0.5 |
| F8* | Chlorhexidine | 3 | 25x diluted | 2 | < 0.001 | 0.004 | 0.004 | 0.125 |
| F8 | Chlorhexidine | 5,8 | 25x diluted | 2 | < 0.001 | 0.004 | 0.004 | 0.125 |
| F8 | Chlorhexidine | 10 | 25x diluted | 2 | < 0.001 | 0.004 | 0.002 | 0.125 |
| F8 | Chlorhexidine | 12,8 | 25x diluted | 2 | < 0.001 | 0.004 | 0.002 | 0.125 |
| F8 | Chlorhexidine | 16,8 | 25x diluted | 2 | < 0.001 | 0.004 | 0.002 | 0.125 |
| F8* | Chlorhexidine | 21 | 25x diluted | 2 | < 0.001 | 0.004 | 0.002 | 0.125 |
| F8* | Chlorhexidine | 24,8 | 25x diluted | 4 | < 0.001 | 0.004 | 0.004 | 0.125 |
| F8 | Chlorhexidine | 28 | 25x diluted | 4 | < 0.001 | 0.004 | 0.002 | 0.125 |
| F8 | Chlorhexidine | 31 | 10x diluted | 4 | < 0.001 | 0.004 | 0.002 | 0.125 |
| F8 | Chlorhexidine | 33 | 25x diluted | 2 | < 0.001 | 0.004 | 0.002 | 0.125 |
| F9* | Chlorhexidine | 3 | 25x diluted | 2 | < 0.001 | 0.004 | 0.002 | 0.125 |
| F9 | Chlorhexidine | 5,8 | 25x diluted | 2 | < 0.001 | 0.004 | 0.002 | 0.125 |
| F9 | Chlorhexidine | 10 | 25x diluted | 2 | < 0.001 | 0.004 | 0.002 | 0.125 |
| F9 | Chlorhexidine | 12,8 | 25x diluted | 2 | < 0.001 | 0.004 | 0.004 | 0.125 |
| F9 | Chlorhexidine | 16,8 | 25x diluted | 2 | < 0.001 | 0.004 | 0.004 | 0.125 |
| F9 | Chlorhexidine | 21 | 25x diluted | 2 | < 0.001 | 0.004 | 0.004 | 0.125 |
| F9 | Chlorhexidine | 24,8 | 25x diluted | 2 | < 0.001 | 0.004 | 0.004 | 0.125 |
| F9 | Chlorhexidine | 28 | 25x diluted | 2 | < 0.001 | 0.004 | 0.004 | 0.125 |
| F9* | Chlorhexidine | 31 | 25x diluted | 2 | < 0.001 | 0.004 | 0.004 | 0.125 |
| F9* | Chlorhexidine | 33 | 25x diluted | 4 | < 0.001 | 0.004 | 0.004 | 0.125 |
| F10 | Chlorhexidine | 3 | 25x diluted | 2 | < 0.001 | 0.004 | 0.002 | 0.125 |
| F10 | Chlorhexidine | 5,8 | 25x diluted | 2 | < 0.001 | 0.004 | 0.002 | 0.125 |
| F10 | Chlorhexidine | 10 | 25x diluted | 2 | < 0.001 | 0.004 | 0.004 | 0.125 |
| F10 | Chlorhexidine | 12,8 | 25x diluted | 2 | < 0.001 | 0.004 | 0.004 | 0.125 |
| F10 | Chlorhexidine | 16,8 | 25x diluted | 2 | < 0.001 | 0.004 | 0.002 | 0.125 |
| F10 | Chlorhexidine | 21 | 25x diluted | 2 | < 0.001 | 0.004 | 0.004 | 0.125 |
| F10 | Chlorhexidine | 24,8 | 25x diluted | 2 | < 0.001 | 0.004 | 0.004 | 0.125 |
| F10* | Chlorhexidine | 28 | 25x diluted | 2 | < 0.001 | 0.004 | 0.004 | 0.125 |
| F10* | Chlorhexidine | 31 | 10x diluted | 8 | < 0.001 | 0.004 | 0.004 | 0.125 |
| F10 | Chlorhexidine | 33 | 25x diluted | 8 | < 0.001 | 0.004 | 0.004 | 0.25 |
| F10 | Chlorhexidine | 37 | 25x diluted | 2 | < 0.001 | 0.004 | 0.004 | 0.125 |
| F10* | Chlorhexidine | 40 | 25x diluted | 20 | < 0.001 | 0.015 | 0.004 | 0.25 |
| F11* | Chlorhexidine | 3 | 25x diluted | 2 | < 0.001 | 0.004 | 0.002 | 0.125 |
| F11 | Chlorhexidine | 5,8 | 25x diluted | 2 | < 0.001 | 0.004 | 0.002 | 0.125 |
| F11 | Chlorhexidine | 10 | 25x diluted | 2 | < 0.001 | 0.004 | 0.002 | 0.125 |
| F11 | Chlorhexidine | 12,8 | 25x diluted | 2 | < 0.001 | 0.004 | 0.004 | 0.125 |
| F11* | Chlorhexidine | 16,8 | 25x diluted | 2 | < 0.001 | 0.004 | 0.002 | 0.125 |
| F11* | Chlorhexidine | 21 | 25x diluted | 4 | < 0.001 | 0.004 | 0.004 | 0.125 |
| F11 | Chlorhexidine | 24,8 | 25x diluted | 4 | < 0.001 | 0.004 | 0.004 | 0.125 |
| F11 | Chlorhexidine | 28 | 25x diluted | 2 | < 0.001 | 0.004 | 0.004 | 0.125 |
| F11 | Chlorhexidine | 31 | 25x diluted | 8 | < 0.001 | 0.008 | 0.004 | 0.125 |
| F11 | Chlorhexidine | 33 | 25x diluted | 20 | < 0.001 | 0.015 | 0.004 | 0.25 |
| F11* | Chlorhexidine | 37 | 25x diluted | 8 | < 0.001 | 0.008 | 0.004 | 0.25 |
| F11* | Chlorhexidine | 40 | 25x diluted | 20 | 0.002 | 0.06 | 0.004 | 0.25 |
| Control1 | Control | 3 | 25x diluted | 2 | < 0.001 | 0.004 | 0.002 | 0.125 |
| Control1 | Control | 33 | 25x diluted | 2 | < 0.001 | 0.004 | 0.004 | 0.125 |
| Control2 | Control | 3 | 25x diluted | 2 | < 0.001 | 0.004 | 0.002 | 0.125 |
| Control2 | Control | 33 | 25x diluted | 2 | < 0.001 | 0.004 | 0.002 | 0.125 |
| Original | Reference strain | 0 | 25x diluted | 2 | < 0.001 | 0.004 | 0.002 | 0.125 |

Data used for analysing correlations is highlighted in yellow. Strains sent for sequencing are indicated with an *.
